# Guidance on the Conduct of Clinical Research within OECD Countries during the Early Stages of the COVID-19 Pandemic: A Systematic Review

**DOI:** 10.3390/pharmacy11010015

**Published:** 2023-01-12

**Authors:** Renu Bhutkar, Jack C. Collins, Claire L. O’Reilly, Sarira El-Den

**Affiliations:** The University of Sydney School of Pharmacy, Faculty of Medicine and Health, The University of Sydney, Sydney, NSW 2006, Australia

**Keywords:** COVID-19, guideline, pandemics, research, research design, systematic review

## Abstract

Background: In response to the COVID-19 pandemic, member countries of the Organisation for Economic Co-operation and Development (OECD) rapidly published guidance regarding the conduct of clinical research. A systematic review was conducted to explore the recommendations issued in relation to the commencement, continuation and termination of clinical research during the early phases of the pandemic. Methods: Searches consisting of the terms “COVID-19”, “clinical research”, and “guidance”, were conducted in PubMed, Embase, MEDLINE, Trip, Guidelines International Network, and Google in April–May 2021 (up to 4 May 2021). Data were extracted from guidance published from OECD member countries and mapped to inductively-developed categories. Results: 9419 references were systematically screened, resulting in the inclusion of 46 publications from 27 OECD countries. Thirty-three sources made recommendations regarding monitoring, risk-benefit assessments and information technology. There was limited specific recommendations made in relation to personal protective equipment (PPE) in the included guidance. Findings demonstrate that guidance differed by publication date demonstrating the rapidly evolving environment within which research was conducted. Importantly, many organisations opted to endorse existing guidance published by the United States’ Food and Drug Administration and the European Medicines Agency rather than develop their own recommendations. Conclusions: Given the rapidly evolving nature of the pandemic, particularly in the early stages, findings demonstrate the global response in relation to clinical research conduct, thereby providing important insights for future public health emergencies.

## 1. Introduction

The novel Coronavirus Disease 2019 (COVID-19) rapidly spread across the world, upending the day-to-day lives of individuals since it was declared a pandemic by the World Health Organisation (WHO) in March, 2020 [1]. Due to the transmission and associated morbidity and mortality of COVID-19, government-mandated safety measures, such as travel restrictions [2] and social distancing requirements [3], the pandemic has resulted in a plethora of changes, which have impacted work environments, including the conduct of clinical research [4]. The National Institutes of Health defines clinical research as research that is conducted with human subjects that is patient-orientated [5]. The Organisation for Economic Co-operation and Development (OECD) is an international organisation comprised of 38 high-income member countries which predominantly have the most research output [6]. In response to the ongoing disruption caused by the COVID-19 pandemic, institutions and organisations within member countries of the OECD released guidance related to the commencement, continuation and termination of clinical research. Guidance is crucial when responding to a public health emergency such as a global pandemic, to prevent loss of life and protect national bodies [7]. Consequences of ill-preparedness for previous epidemics and pandemics, such as Influenza A H1N1 2009 (Swine Flu) and Ebola outbreaks have been well documented and demonstrate the importance of consistent guidance that is distributed to the public [7]. There is a need to systematically identify, synthesise and comparatively analyse these recommendations to understand how clinical research was impacted during the early stages of the pandemic. While previous studies have published the impact of the COVID-19 pandemic on clinical research such as risk-benefit ratios of trials, ethical principles of research, technology use and remote monitoring, there has been no comparative analysis of guidance published during the early stages of the pandemic, which demonstrates the gaps in knowledge present within the literature during such unprecedented times. The aim of this systematic review was to identify, synthesise, and comparatively analyse guidance issued by OECD member countries relating to the conduct of clinical research during the early stages of the COVID-19 pandemic. This comparative synthesis demonstrates the strengths and limitations of recommendations and beneficial principles that can be carried forward to guidance published during future pandemics. 

## 2. Materials and Methods

### 2.1. Search Strategy

A systematic literature search guided by the PRISMA guidelines [8] was conducted in consultation with an academic librarian within PubMed, Embase, MEDLINE, Trip and the Guidelines International Network (GIN) between 1 January 2020 to 28 April 2021 (Supplementary File S1). Three main terms relating to “COVID-19”, “clinical research” and “guidance” were used in the search, whereby subject headings were mapped for each term where possible, depending on the database.

The World Wide Web via Google search engine was also systematically searched on 4 May 2021, for relevant recommendations issued from the 37 OECD member countries at the time of the search. The following search was repeated 37 times in Google, each time with the addition of the country name of each of the 37 OECD member countries:

(*COVID* OR *coronavirus*) AND (*guidance* OR *guideline* OR *recommendation*) AND (*RCT* OR *“clinical trial” OR “controlled trial”*) AND *country*.

The region settings were changed within Google to search within each of the 37 OECD countries. Browsing was conducted using “Incognito mode”, to minimise the risk of results being personalised based on location, previous searches or HTTP cookies saved on the browser. The first 40 results were taken from each of the 37 searches (when available).

The systematic search results from each database, registry and search engine were imported to EndNote (databases) or to an Excel spreadsheet (registry, search engine). Potentially relevant publications were also identified through website searching, citation tracking, and reference chaining during the title screening stage. 

### 2.2. Selection Criteria

After automatic and manual duplicate removal, systematic screening by title, abstract and full-text was conducted by a primary reviewer (RB). A secondary reviewer (JCC) completed a random audit of 25% of the excluded records. Disagreements were resolved through discussion and, when necessary, involved a third member of the research team (SE-D) to reach consensus. 

For inclusion, publications had to provide explicit guidance or recommendations pertaining to the conduct of clinical research. Included publications:

1. provided guidance on conducting clinical research during the COVID-19 pandemic;
2. were authored by a body (e.g., organisation, governing body, institution) based in a member country of the OECD;
3. were available in English; and
4. were published online.

Included publication types included official guidance documents, recommendations posted on national government websites, guidance from independent organisations as well as clinical research websites that endorsed other guidance. Publications that were the opinions of individuals published as commentaries and news articles, as well as those published through personal websites (e.g., blogs), legal consultancy firms, and private pharmaceutical companies were excluded. 

### 2.3. Data Extraction

A guidance extraction matrix was inductively developed through an iterative process, consisting of multiple discussions with all reviewers to refine. It comprised of nine categories: (1) Monitoring (2) Risk-benefit assessments and contingency measures (3) COVID-19 Symptom Screening (4) Continuation of Trials (5) Recruitment of Participants (6) Remote Source Data Verification (SDV) (7) Information Technology (IT) (8) Delivery of Investigational Medicinal Product (IMP) (9) Informed Consent. Publications which endorsed pre-existing guidance, but did not issue their own guidance, were tabulated separately. Where one source provided multiple guidance documents in one medium (e.g., website), these were merged in the tables, where necessary.

## 3. Results

### 3.1. Search Results and Included Studies

**Figure 1:**
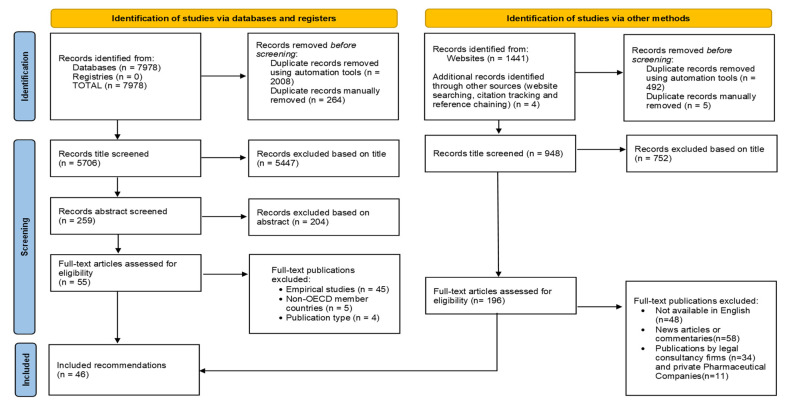
Flow diagram of screening process [8].

The database and registry searches produced 7978 potentially relevant publications, of which 2272 were duplicates. After title, abstract and full-text screening, another 5447, 204 and 54 references were excluded, respectively (Figure 1).

The Google search produced 1441 potentially relevant publications. Results from each of the 37 searches were manually imported into Excel for 35/37 countries (two searches produced only 34 and seven results). After deduplication, title and full-text screening, 46 were considered eligible for inclusion (Figure 1).

### 3.2. General Characteristics

**Figure 2:**
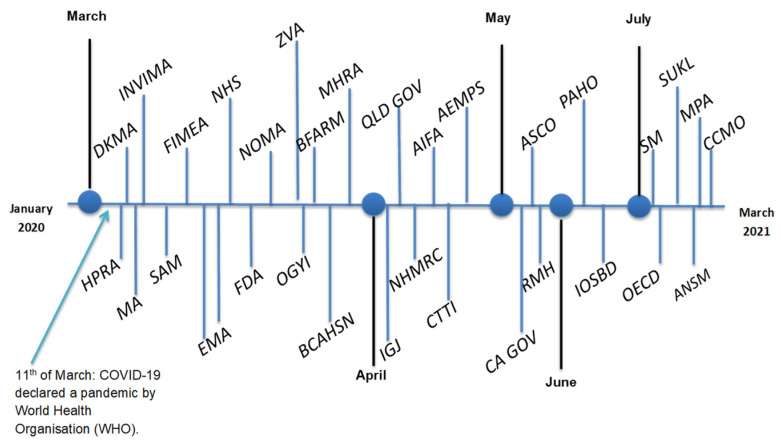
Dates of publication of included publications.

Forty-six publications from 27 OECD member countries were included, whereby most were issued by Australia (*n* = 8), Canada (*n* = 8) and the United States (*n* = 6). Guidance was published on websites (*n* = 27) and in guidance documents (*n* = 14), position statements (*n* = 2), journal articles (*n* = 2) and an opinion piece (*n* = 1). Forty-five publications were originally published between 12 March 2020 and 23 February 2021. The original date of publication for one publication was not provided [9]. Figure 2 provides a visual representation of publication dates.

Thirty-four publications (from 33 sources) reflected recommendations developed by a body within an OECD member country (Table 1), while 12 publications endorsed existing guidance by another body, only (Table 2).

### 3.3. Findings from Publications That Developed Recommendations (n = 33)

Table 1 presents the different recommendation domains developed to guide the conduct of clinical research during the COVID-19 pandemic within each of the 34 publications, whereby one publication exclusively focused on informed consent [14].

### 3.4. Monitoring

Alternative monitoring of participants was recommended in most publications, whereby remote monitoring visits were encouraged by multiple publications, to reduce the burden on hospitals and to ensure adherence to social distancing regulations [10,11,13,15,16,22,26,32,35,37,39,42]. Other recommendations included extending the interval of on-site monitoring, postponing of monitoring visits, and transitioning from in-person monitoring to telephone/video calls or emails instead. Centralised monitoring through electronic data capture systems such as electronic case report forms [17,19,25,31,34] and electronic patient reported outcomes [19] was also recommended in publications. Publications also noted the importance of establishing follow-up measures and planning for ongoing and increased monitoring, after the situation normalised.

### 3.5. Risk-Benefit Assessment and Contingency Measures

While conducting a risk-benefit assessment was discussed in 22 publications [9,13,14,15,17,18,19,22,23,25,26,27,28,29,32,33,34,35,36,38,39,40,43], it was only actively advocated for in 16 out of those 22, endorsing that risks to participants and staff should be carefully balanced with patient safety and preservation of data integrity. High-risk patient populations, such as those who were taking corticosteroids or immunosuppressants, over the age of 60 or had multiple comorbidities, were to be considered when recruiting participants [9,25,26,28,32,35,40]. Furthermore, risk-benefit assessments were recommended when contingency planning for alternative procedures, so that rapid responses could be implemented if needed. 

### 3.6. COVID-19 Symptom Screening

Eight publications [9,11,12,16,20,37,38,40] encouraged screening participants for COVID-19 symptoms at research facilities. This involved asking patients whether they were experiencing any flu-like symptoms and if they had been a close contact of COVID-19 recently before entering a research facility.

### 3.7. Continuation of Clinical Research

Two publications [18,19] advised that trials that had not started yet should be postponed, emphasising that the safety of the participants was paramount. Fifteen publications [9,11,12,16,17,18,19,22,28,29,30,34,38,39,43] recommended that in-depth assessment was required by sponsors and primary investigators (PIs) to determine whether ongoing clinical research should continue. Six publications [16,17,19,26,41,43] recommended prioritising COVID-19 treatment and prevention trials. 

### 3.8. Recruitment of Participants

While 18 publications [9,10,13,15,16,17,18,19,24,25,26,28,29,31,32,34,35] encouraged participant recruitment if risk-assessments were carried out, publications by Hungary’s National Institute of Pharmacy and Nutrition (OGYI) [25], Ireland’s Health Products Regulatory Authority (HPRA) [26], and Germany’s Federal Institute for Drugs and Medical Devices (BFARM) [24] actively discouraged the recruitment of any participants.

### 3.9. Remote Source Data Verification

As can be seen in Table 1, remote source data verification (SDV) was endorsed by multiple publication, but was also discouraged by other publications, such as those from Estonia [18], Norway [31], and The Netherlands [30], with some citing that it jeopardises trial participants’ privacy rights. Those that did endorse remote SDV, highlighted the importance of confidentiality and consent.

### 3.10. Use of Information Technology (IT)

Switching to technological means of communication, to reduce the risk of infection through in-person meetings, was commended by 30 out of 33 sources. Using technology to conduct teletrials, meet through videoconferencing, conduct remote audits, obtain digital consent, and monitor patients through mobile applications were some examples of recommendations made, whereby the need for security measures to prevent unauthorised data access, maintain data backup and destroy redacted data and documentation was noted.

### 3.11. Delivery and Administration of Invetigational Medicinal Product (IMP)

Twenty-seven publications [9,11,13,15,16,17,18,19,21,22,23,25,26,27,28,29,30,31,32,33,34,36,37,38,39,40,42] noted the possibility of delivering IMPs from the site to participants’ homes, thereby allowing quarantined or locked-down participants to participate in research, ensuring project continuity while decreasing the risk of infection spread. The need to obtain consent from participants for providing their home address was noted, as was the importance of training subjects to administer IMPs, ensuring IMPs’ stability during transport and storage, and appropriately and safely disposing of used IMPs. Five publications [17,27,36,39,40] made specific recommendations regarding the safe administration of IMPs in participants’ homes. Suggestions included providing participants with drug disposal pouches and requesting participants to provide documentation (e.g., photos, videos) when disposing IMPs. Nine publications [16,17,18,19,25,26,27,32,33] provided recommendations pertaining to the supply of large amounts of IMPs and for periods longer than necessary, in case of a lockdown or a stock shortage.

### 3.12. Ethical Considerations and Informed Consent

Approximately half of the publications in Table 1 recommended modifying or reconsidering consent processes, such as by asking participants who were already participating in ongoing research to consent again due to new restrictions resulting in changes to the conduct of clinical research [9,11,14,16,19,25,26,30,31,32,34,37,38,39,41,42]. The publication by the British Columbia Academic Health Science Network (BCAHSN) [14], solely focused on informed consent and noted the importance of electronic methods of consenting, to avoid contamination and training staff on new consenting procedures. Publications recommended that PIs educate participants on the potential risks and benefits associated with participating in research in the context of the pandemic and allow participants adequate time to ask questions. Virtual and electronic methods of obtaining consent were also discussed, reinforcing the importance of avoiding unnecessary in-person contact while adhering to ethical principles. Increased confidentiality measures such as remote monitoring, whereby firewalls, secure log-ins, and strict rules on sharing patient documents were recommended. 

### 3.13. Findings from Publications Endorsing Other Recommendations

Twelve publications, tabulated in Table 2, were identified that endorsed recommendations made by other bodies (presented in Table 1). A majority of these were websites (*n* = 11) as well as one position statement (*n* = 1) and were obtained from research organisations, university laboratory websites and government websites. Of these 12 publications, five endorsed the guidance of the NHMRC and Health Canada, while four endorsed the FDA and one endorsed the EMA guidance.

### 3.14. Quality of Included Guidance

As publication types included in this review were varied to allow for a comprehensive overview of recommendations, assessment with formal quality appraisal tools was not considered appropriate or feasible; however, the quality of included publications was considered. Of the 46 included publications, much of the recommendations were based on guidance published by the FDA [39] and the EMA [19]. This may be because these publications were the most thorough, covering nearly all recommendation domains (Table 1). However, pandemic experiences have been different across countries. For example, during the Delta-strain outbreak, the US reported a maximum of 251,084 new confirmed cases per day [55], while Australia had not exceeded 239 cases per day at that time [56]. Therefore, there is a need to ensure that guidance is targeted to the context in which it is intended to be applied. Publications by the EMA and FDA had minimal references, which can be attributed to the unprecedented nature of the pandemic. All 33 original recommendation sources were from bodies that were reputable, provided relevant contact details for any discrepancies to be ameliorated and 33 out of 34 publications did not contain any conflict of interests. One publication by the American Society of Oncology Trials [42], was reported to be written by authors sponsored by pharmaceutical companies. However, the publication itself was not sponsored by these companies and it made similar recommendations to its counterparts. 

## 4. Discussion

The COVID-19 pandemic considerably impacted clinical research and the way researchers work, especially in the early phases of the pandemic when there was a need to operate in accordance with government-mandated policies. While there are multiple publications describing the effect of the COVID-19 pandemic on clinical research or specific areas of research, this review is the first to systematically identify and synthesise the recommendations issued early on in the pandemic to guide the conduct of clinical research, during a period of unprecedented uncertainty and rapid adaptation. In general, most publications identified in this review made recommendations relating to remote monitoring measures, the use of technology, delivery of IMPs, completion of risk-benefit assessments, and changes to informed consent procedures. These categories, commonly identified in recommendations from around the world, demonstrate what is most important to consider when advising researchers conducting projects during a time of disruption and instability. 

### 4.1. Differences in Guidance Based on Date Published

Due to the urgent nature of the pandemic, the majority of included publications were published during the months of March and April 2020 which was foreseeable as COVID-19 was declared a pandemic by WHO on 11 March 2020 [57]. The earliest guidance was published 24–48 h following the declaration, by the UK Health Research Authority (NHS) [37], the Danish Medicines Agency (DKMA) [17], and Ireland’s HPRA [26]. This systematic review demonstrates that guidance differed in the breadth of topics covered regarding different aspects of clinical research depending on the date it was published, showing that guidance published earlier in the pandemic was not as comprehensive, compared to guidance published in the second-half of 2020. Furthermore, recommendations published 24 h to two weeks following the declaration, such as guidance from the NHS [37] and BFARM [24] did not include recommendations regarding IMP delivery, risk-benefit assessment, contingency planning, and monitoring, which were commonly identified domains in recommendations published later on, as the pandemic evolved. In fact, guidance released by these organisations during the early stages of the pandemic did not provide any recommendations regarding the continuity of trials, possibly due to the uncertain nature of the pandemic. Moreover, early recommendations by BFARM [24], OGYI [25] and HPRA published in March 2020, discouraged any recruitment of participants. This can be placed in comparison to guidance published a month later, such as recommendations by the Australian NHMRC [10] and the US Clinical Trials Transformation Initiative [38], which made a broader range of recommendations, in general, and did not advise against continuity of research or recruitment of participants, so long as risk mitigation strategies were put in place. These are important findings for researchers planning future projects, as many granting bodies require researchers to provide details surrounding risk assessment and mitigation. Throughout the last two years of the pandemic, the spread of COVID-19 has drastically increased in some countries, whereby changes to virology have necessitated an evolution of attitudes and government policies. Similar to experiences in the education sector [58], the initial guidance identified in this review was ‘emergency-based’, but as time went on, researchers had to shift their mindframe from that of halting or pausing research early on in the pandemic, to finding ways to safely ensure the continuation of research, once it became evident that the pandemic was unlikely to be a short-lived disruption. As the response to the pandemic evolved with the development of COVID-19 vaccines and their rollout, this also presented barriers to researchers’ ability to physically return to workplaces and guidance surrounding the conduct of clinical research. Therefore, it is imperative to not only monitor how guidance changes, but also ensure that guidance reflects the changing values and pandemic environment, worldwide.

### 4.2. Limited Discussion of PPE

None of the included publications made recommendations regarding PPE use in the context of conducting research, specifically, although this term has become part of the public’s vocabulary [59]. Eight publications recommended the use of infection control measures such as disposal of biohazard samples [10,39] and conducting COVID-19 symptom screening [9,11,12,16,20,42]. Some of the main methods of COVID-19 infection control have included the use of masks, gloves and washing/sanitising hands regularly [60]. An explanation for this could be that guidance for PPE and infection control measures was delegated to local health authorities and was already inherently embedded into the everyday lives of the public. This could have also been due to the unpredictability of the situation, whereby levels of PPE enforcement and infection control supplies were constantly changing in different locations. 

### 4.3. Principles to Be Carried Forward and Potential Long-Term Impact of Recommendations

Emerging literature is demonstrating how the pandemic may impact patients’ clinical outcomes. Early in the pandemic, there were fears that decreased patient recruitment and a lack of in-person scientific collaboration would pose challenges to the conduct clinical research. For example, the pandemic resulted in reduced activation of cancer clinical trials in 2020 especially in the US where estimations of trial activations were only 57% of what was anticipated [61]. Similarly, a reduction in prostate, lung, bladder and colorectal cancer screening also resulted, potentially impacting diagnosis and treatment of these cancers [62].

On the other hand, there were beneficial principles introduced in this unprecedented situation that could be carried forward. For example, researchers learnt how to optimise the use of remote SDV, conduct teletrials, and deliver IMPs, and these activities may prove beneficial beyond the pandemic. However, there are also drawbacks to these methods, whereby the delivery of IMPs to participants’ homes does not ensure correct administration and/or storage, remote SDV can increase the risk of patient confidentiality violations and remote monitoring can compromise the integrity of clinical research. Nonetheless, clinical researchers have come up with innovative ways to conduct research during the most difficult of times, through the use of technology, and the flexibility and adaptability that was required to ensure the continuation of clinical research during the pandemic may allow clinical research to become resilient to future stressors [63].

### 4.4. Strengths and Limitations of the Review

Despite several strengths, including a robust search strategy and systematic search of databases and Google which enabled the identification and synthesis of relevant guidance, potential limitations of this systematic review need to be considered. Only guidance available in English and published online was included in this systematic review, resulting in potentially informative guidance, especially from Asian, South American and European countries, being excluded. Furthermore, only guidance issued by member countries of the OECD was included, excluding guidance from countries such as China and India with high research output [64]. Nonetheless, as the first publication to systematically review the evidence in this area, it paves the way for future research in this area. 

## 5. Conclusions

This systematic review provides a comprehensive overview of the guidance published relating to the commencement, continuation and termination of clinical research in OECD member-countries during the early stages of the unprecedented and challenging COVID-19 pandemic. This systematic review demonstrates the rapid response delivered by OECD member-countries to publish guidance that ensured the safe conduct of clinical research across settings, and how the response evolved alongside the evolution of the pandemic and our response. Guidance should be appropriately updated as new evidence emerges and the pandemic evolves. Findings may contribute to the development of guidance in future public health emergencies.

## Figures and Tables

**Table 1:** Summary of publications that developed recommendations.

Abbreviation of Publishing Body	Country of Origin	Date of Publication/Update	Monitoring	Risk-Benefit Assessment and Contingency Measures	COVID-19 Symptom Screening	Continuation of Trials	Recruitment of Participants	Remote Source Data Verification	Information Technology	Delivery of IMP to Participant Home	Informed Consent
NHMRC * [10]	AUSTRALIA	9 April 2020	**✓**	**✓**	**N/R**	**✓**	**✓**	**✓**	**✓**	**✓**	**N/R**
**QLD GOV** [11]	AUSTRALIA	7 April 2020	**✓**	**✓**	**✓**	**✓**	**N/R**	**N/R**	**✓**	**✓**	**N/R**
**RMH** [12]	AUSTRALIA	13 May 2020	**X**	**✓**	**✓**	**N/R**	**N/R**	**N/R**	**✓**	**N/R**	**N/R**
MA ** [13]	AUSTRALIA	23 March 2020	**✓**	**N/R**	**N/R**	**N/R**	**✓**	**N/R**	**✓**	**✓**	**N/R**
**BCAHSN** [14]	CANADA	March 2020	**X**	**✓**	**N/R**	**N/R**	**N/R**	**N/R**	**N/R**	**N/R**	**✓**
**CA GOV** [9]	CANADA	Updated 6 May 2021	**✓**	**✓**	**✓**	**N/R**	**✓**	**N/R**	**✓**	**✓**	**✓**
**INVIMA** [15]	COLOMBIA	17 March 2020	**✓**	**✓**	**N/R**	**✓**	**✓**	**N/R**	**✓**	**✓**	**N/R**
**SUKL** [16]	CZECH REPUBLIC	22 December 2020	**✓**	**N/R**	**✓**	**✓**	**✓**	**N/R**	**✓**	**✓**	**✓**
**DKMA** [17]	DENMARK	13 March 2020	**✓**	**✓**	**N/R**	**✓**	**✓**	**✓**	**✓**	**✓**	**N/R**
**SAM** [18]	ESTONIA	18 March 2020	**✓**	**✓**	**N/R**	**N/R**	**✓**	**X**	**✓**	**✓**	**N/R**
**EMA** [19]	EUROPEAN UNION	25 March 2020	**✓**	**✓**	**N/R**	**✓**	**✓**	**✓**	**✓**	**✓**	**✓**
**EMA** [20]	EUROPEAN UNION	25 March 2020	**✓**	**✓**	**✓**	**✓**	**✓**	**N/R**	**N/R**	**N/R**	**N/R**
**FIMEA** [21]	FINLAND	19 March 2020	**✓**	**N/R**	**N/R**	**N/R**	**N/R**	**N/R**	**✓**	**✓**	**N/R**
**ANSM** [22]	FRANCE	21 October 2020	**✓**	**✓**	**N/R**	**✓**	**N/R**	**N/R**	**✓**	**✓**	**N/R**
**OECD** [23]	INTERNATIONAL ORGANISATION	4 August 2020	**N/R**	**N/R**	**N/R**	**N/R**	**N/R**	**N/R**	**✓**	**✓**	**N/R**
**BfArM** [24]	GERMANY	30 March 2020	**N/R**	**N/R**	**N/R**	**N/R**	**X**	**N/R**	**✓**	**N/R**	**N/R**
**OGYI** [25]	HUNGARY	25 March 2020	**N/R**	**✓**	**N/R**	**N/R**	**X**	**N/R**	**✓**	**✓**	**✓**
**HPRA** [26]	IRELAND	13 March 2020	**✓**	**✓**	**N/R**	**N/R**	**X**	**N/R**	**✓**	**✓**	**✓**
**AIFA** [27]	ITALY	7 April 2020	**N/R**	**✓**	**N/R**	**✓**	**N/R**	**N/R**	**✓**	**✓**	**✓**
**ZVA** [28]	LATVIA	17 March 2020	**N/R**	**N/R**	**N/R**	**✓**	**✓**	**N/R**	**✓**	**✓**	**N/R**
**CCMO** [29]	NETHERLANDS	23 February 2021	**N/R**	**N/R**	**N/R**	**✓**	**✓**	**X**	**✓**	**✓**	**✓**
**IGJ** [30]	NETHERLANDS	8 April 2020	**✓**	**N/R**	**N/R**	**✓**	**N/R**	**X**	**N/R**	**✓**	**✓**
**NOMA** [31]	NORWAY	16 March 2020	**✓**	**N/R**	**N/R**	**N/R**	**✓**	**X**	**✓**	**✓**	**✓**
**AEMPS** [32]	SPAIN	27 April 2020	**✓**	**✓**	**N/R**	**N/R**	**✓**	**N/R**	**✓**	**✓**	**✓**
**MPA** [33]	SWEDEN	18 December 2020	**✓**	**✓**	**N/R**	**N/R**	**N/R**	**✓**	**✓**	**✓**	**N/R**
**SM** [34]	SWITZERLAND	17 December 2020	**N/R**	**✓**	**N/R**	**✓**	**✓**	**✓**	**✓**	**✓**	**✓**
**MHRA** [35,36]	UK	19 March 2020	**✓**	**✓**	**N/R**	**✓**	**✓**	**N/R**	**✓**	**✓**	**N/R**
**NHS** [37]	UK	12 March 2020	**✓**	**N/R**	**✓**	**✓**	**✓**	**✓**	**✓**	**N/R**	**✓**
**CTTI** [38]	USA	13 April 2020	**N/R**	**✓**	**✓**	**✓**	**✓**	**N/R**	**✓**	**✓**	**✓**
**FDA** [39]	USA	18 March 2020	**✓**	**✓**	**N/R**	**N/R**	**N/R**	**N/R**	**✓**	**✓**	**✓**
**IOSIBD** [40]	USA	10 June 2020	**N/R**	**N/R**	**✓**	**✓**	**✓**	**N/R**	**✓**	**✓**	**N/R**
**PAHO** [41]	USA	5 June 2020	**✓**	**N/R**	**N/R**	**✓**	**N/R**	**N/R**	**✓**	**N/R**	**N/R**
**ASCO** [42]	USA	12 May 2020	**N/R**	**N/R**	**N/R**	**N/R**	**N/R**	**N/R**	**✓**	**✓**	**N/R**

√: advised by publication; X: discouraged by publication; N/R: category not reported. * The document was hosted on the NHMRC website, but authored by All state and Territory Departments of Health, Clinical Trials Project Reference Group, National Health and Medical Research Council, and the Therapeutic Goods Administration. ** MA, Ausbiotech and Medical Technology Association as part of the Research & Development Taskforce. Abbreviations: AEMPS: Spanish Agency of Medicines and Medical Devices (Spain); AIFA: Italian Medicines Agency (Italy); ANSM: National Agency for the Safety of Medicines and Health Products (France); ASCO: American Society of Clinical Oncology (United States); BCAHSN: British Columbia Academic Health Science Network (Canada); BfArM: Federal Institute for Drugs and Medical Devices (Germany); CA GOV: Canada Government (Canada); CCMO: Central Committee on Research Involving Human Subjects (The Netherlands); CTTI: Clinical Trials Transformation Initiative (United States); DKMA: Danish Medicines Agency (Denmark); EMA: European Medicines Agency (European Union); FDA: Food and Drug Administration (United States); FIMEA: Finnish Medicines Agency (Finland); HPRA: Health Products Regulatory Authority (Ireland); IGJ: Health and Youth Care Inspectorate (The Netherlands); INVIMA: National Food and Drug Surveillance Institute (Colombia); IOSBD: International Organisation for the Study of Inflammatory Bowel Disease (United States); MA: Medicines Australia (Australia); MHRA: Medicines and Healthcare products Regulatory Agency (United Kingdom); MPA: Swedish Medical Products Agency (Sweden); NHMRC: National Health and Medical Research Council (Australia); NHS: Health Research Authority (United Kingdom); NOMA: Norwegian Medicines Agency (Norway); OECD: Organisation for Economic Cooperation and Development (International Organisation); OGYI: National Institute of Pharmacy and Nutrition (Hungary); PAHO: Pan American Health Organisation (International Organisation); QLD GOV: Queensland Government (Australia); RMH: The Royal Melbourne Hospital (Australia); SAM: State Agency of Medicines (Estonia); SM: SwissMedic (Switzerland); SUKL: State Institute for Drug Control (Czech Republic); UK: United Kingdom; USA: United States of America; ZVA: State Agency of Medicines Republic of Latvia (Latvia).

**Table 2:** Endorsement of published recommendations.

	Country	Name of Document/Website	Body	Endorsed Recommendation
1	Australia	COVID-19: Guidance on Australian clinical trials for institutions, HRECs, researchers and sponsors [43]	ACTA	NHMRC
2	Australia	Clinical Trial Processes [44]	TGA	NHMRC
3	Australia	COVID-19 clinical trial guidance [45]	NSW Health	NHMRC
4	Australia	Clinical trial conduct during the COVID-19 pandemic [46]	CTIQ	NHMRC
5	Belgium	Responsibility of Research Ethics Committees during the COVID-19 Pandemic [47]	EUREC	EMA
6	Canada	Guidance during COVID-19 Outbreak [48]	HREBA	FDA Health Canada
7	Canada	Health Canada Notice: Management of clinical trials during COVID-19 [49]	CAPRA	Health Canada
8	Canada	Regulations, guidelines, important links: Quality assurance for clinical trials [50]	UCAL	FDA
9	Canada	Clinical trials or studies involving a drug, medical device, or natural health product [51]	UWaterloo	Health Canada
10	Canada	COVID-19 Resources: Clinical trials and research involving humans [52]	CIHR	Health Canada
11	Canada	COVID-19 Regulatory Updates for the Clinical Trials Community [53]	CTO	Health Canada FDA
12	New Zealand	COVID-19 Resources for Researchers [54]	HSRI	NHMRC FDA

Abbreviations: ACTA: Australian Clinical Trials Alliance (Australia); CAPRA: Canadian Association of Professionals in Regulatory Affairs (Canada); CIHR: Canadian Institute of Health Research (Canada); CTIQ: Clinical Trials (Australia); CTO: Clinical Trials Ontario (Canada); EUREC: European Network of Research Ethics (Belgium); HREBA: Health Research Ethics Board of Alberta (Canada); HSRI: Health Services Research Institution (New Zealand); NSW Health: New South Wales Health (Australia); TGA: Therapeutic Goods Administration (Australia); UCAL: University of Calgary (Canada); UWaterloo: University of Waterloo (Canada).

## Data Availability

Not applicable.

## Supplementary Materials

The following supporting information can be downloaded at: https://www.mdpi.com/article/10.3390/pharmacy11010015/s1, Supplementary File S1—Database search strategy.
