# Supplementary material for: Guidance on the Conduct of Clinical Research within OECD Countries during the Early Stages of the COVID-19 Pandemic: A Systematic Review
Source: Pharmacy (Basel). 2023 Jan 12;11(1):15. doi: 10.3390/pharmacy11010015 (PMC9844363; doi:10.3390/pharmacy11010015)
Supplement: Supplementary file 1 [file pharmacy-11-00015-s001.zip › pharmacy-2041641-supplementary.pdf]

Supplementary File S1 - Database Search Strategy

**Database**	**MEDLINE and Embase**
**Date**	**28/04/2021**
**Strategy**	**#1 AND #2 AND #3**
**#1**	COVID-19 OR (Covid-19 or Covid 19).mp OR corona*.mp OR Pandemics OR Coronavirus OR pandemic.mp
**#2**	exp clinical trial OR ((clinical or control*) and trial*).mp. OR exp Clinical Trials as Topic OR RCT*.MP
**#3**	guideline/ or practice guideline OR guide*.mp. OR recommendation*.mp. OR
**Filters**	English language and yr=”2020-Current”

**Database**	**PubMed**
**Date**	**28/04/2021**
**Strategy**	**#1 AND #2 AND #3**
**#1**	COVID OR COVID-19 OR corona* OR pandemic
**#2**	RCT* OR "randomized control* trial*" OR "clinical trial*" OR "randomised control* trial*"
**#3**	(guidelines or guid* or practice* or practise*)
**Filters**	English language and yr=”2020-Current”

**Database**	**Trip**
**Date**	**28/04/2021**
**Strategy**	**#1 AND #2 AND #3**
**#1**	COVID OR COVID-19 OR corona* OR pandemic
**#2**	RCT* OR "randomized control* trial*" OR "clinical trial*" OR "randomised control* trial*"
**#3**	Guid* OR guideline* OR recommendation* OR practice*
**Filters**	English language and yr=”2020-Current”

**Database**	**G-I-N**
**Date**	**28/04/2021**
**Strategy**	**#1 AND #2**
**#1**	COVID
**#2**	Clinical trial
**Filters**	English language and yr=”2020-Current”
